# Production of Fungal Pigments: Molecular Processes and Their Applications

**DOI:** 10.3390/jof9010044

**Published:** 2022-12-28

**Authors:** Lan Lin, Jianping Xu

**Affiliations:** 1 Medical School, School of Life Science and Technology, Key Laboratory of Developmental Genes and Human Diseases (MOE), Southeast University, Nanjing 210009, China; 2 Department of Biology, McMaster University, Hamilton, ON L8S 4K1, Canada

**Keywords:** fungi, pigment biosynthesis, stress factors, pigmentogenesis, molecular mechanism

## Abstract

Due to the negative environmental and health effects of synthetic colorants, pigments of natural origins of plants and microbes constitute an abundant source for the food, cosmetic, textile, and pharmaceutical industries. The demands for natural alternatives, which involve natural colorants and natural biological processes for their production, have been growing rapidly in recent decades. Fungi contain some of the most prolific pigment producers, and they excel in bioavailability, yield, cost-effectiveness, and ease of large-scale cell culture as well as downstream processing. In contrast, pigments from plants are often limited by seasonal and geographic factors. Here, we delineate the taxonomy of pigmented fungi and fungal pigments, with a focus on the biosynthesis of four major categories of pigments: carotenoids, melanins, polyketides, and azaphilones. The molecular mechanisms and metabolic bases governing fungal pigment biosynthesis are discussed. Furthermore, we summarize the environmental factors that are known to impact the synthesis of different fungal pigments. Most of the environmental factors that enhance fungal pigment production are related to stresses. Finally, we highlight the challenges facing fungal pigment utilization and future trends of fungal pigment development. This integrated review will facilitate further exploitations of pigmented fungi and fungal pigments for broad applications.

## 1. Introduction

Colors constitute an indispensable part of our life. Most living organisms on our planet display certain colored hues through absorption and refraction of specific wavelengths of light [1,2]. All pigments possess conjugated moieties, namely chromophores, that facilitate electronic resonances and mediate energy transfers within and between cells. The energy captured and/or reflected by pigments has been shown to be involved in multiple biological processes, ranging from the utilization of solar energy for metabolic needs and photo-protection for the maintenance of life, to camouflage, as well as mate and pollinator attraction.

Fungi are ubiquitously distributed in both natural and anthropogenic environments, featuring a vast diversity of species, morphology, and pigmentation [1,3,4]. They can be favorable or detrimental to humans, the latter of which involves a range of mycoses, especially in immunocompromised individuals with immunological deficiency, HIV infection, and undergoing organ transplantation or cancer therapy [5,6]. The significant roles of fungal pigments in microbial pathogenesis have been extensively investigated and documented [1,5,6,7,8]. Studies using fungal mutants with altered pigmentation have revealed how these pigments may provide a survival advantage for pathogens in host environments [8], for example, to aid in the evasion of the host immune system. Similarly, confronted with external environmental stresses such as ultraviolet light, irradiation, nutrient limitation, and osmotic pressure, fungi have evolved the mechanism of pigmentogenesis as adaptive means for their survival and dissemination. Indeed, as common secondary metabolites, fungal pigments have likely played an underestimated but key role in cell protection and ecological interactions with other organisms [9].

The ecological roles of fungal pigments in the interactions with abiotic and biotic factors are similarly reflected in their relevance to human health and human welfare. For example, fungal pigments have been used as natural colorants in foods and as nutraceuticals (functional foods), gaining increasing popularity in certain regions and age groups, including among the elderly for healthy aging [10]. Indeed, fungal pigments, secondary metabolites with tremendously diverse structures, have shown a diversity of bioactivities, such as anti-tumor, anti-obesity, cholesterol-lowering and/or anti-atherosclerotic, anti-Alzheimer disease, anti-oxidative, and immunosuppressive activities [1]. Together, these beneficial bioactivities of fungal pigments to humans make them attractive pharmacophores in the drug industry.

As described above, a common application of fungal pigments is as food colorants. Indeed, food colorants have been used by humans for thousands of years. Most colorants in early human history were derived from natural sources. However, over the last two centuries, synthetic colorants have been increasingly used. Unfortunately, a variety of detrimental effects have been linked to synthetic food colorants, including but not limited to attention deficit/hyperactivity in children, allergies, cancer, reproductive disorders, and neurological impairment [10]. Such reports have propelled the exploration of natural alternatives [10,11,12]. These natural alternatives include natural colorants as well as the natural biological processes that produce these colorants. For long-term sustainable development of this industry, the targeted materials and processes should have as few negative impacts on the environment as possible.

Among the many synthetic food colorants are the yellow colorants tartrazine and sunset yellow as well as the red coloring compounds carmine and amaranth [13]. These colorants have been managed and controlled based on regulations issued by the World Health Organization (WHO). However, their acceptability by consumers has been declining, predominantly due to their associations with various health issues and to their long-term persistence in the ecosystem while posing significant concerns to other organisms in the environment. 

In contrast to the pigments derived from plants such as carotenoids and polyketides, fungi represent an inexhaustible source for bio-pigments and are not limited by seasonal or geographic variations. Due to advances in fermentation devices (bioreactors, also called fermentors) as well as downstream bioprocessing, the cost-effective and readily manipulated microbial (including fungal) systems can be implemented for pigment extraction and purification at a large scale [14,15,16]. Fungal pigments have great potential in the industrial and medical settings. 

To help facilitate the research and development of fungal pigments for commercial applications, here we review the molecular bases governing fungal pigment production. Our focus is on how the biosynthesis of pigments are regulated in industrial and medical fungal species and how environmental factors influence pigmentogenesis in fungi. The environmental factors to be discussed include light, UV radiation, ionization radiation, oxidative agents, temperature, osmotic pressure, and nutrient limitations (carbon and nitrogen sources). In combination with the expanding genome sequences, a better understanding of fungal pigment synthesis pathways, their regulations, and how environmental factors influence their production will also help identify novel fungal species capable of producing valuable pigments for further exploration. 

## 2. Pigmented Fungi and Biosynthesis of Pigments

Many fungi can produce pigments. However, most of our knowledge about fungal pigments has been derived from fungi in four genera: *Aspergillus*, *Penicillium*, *Paecilomyces*, and *Monascus* [1,17]. The known fungal pigments are classified into the following broad types: carotenoids, melanins, polyketides, and azaphilones (polyketide derivatives) [1,18]. Below, we describe each of these categories.

### 2.1. Carotenoids

‘Carotene’, derived from the Latin word carota, was coined in 1831 by Wackenroder, who extracted and purified the orange pigment from carrot (*Daucus carota*). The names of carotenoids do not reflect their structural cues but rather are based on the stem name ‘carotene’, which is preceded by Greek prefixes (e.g., α, β, ε) that denote the two end groups [19]. 

The charming colors and the health-promoting effects of various carotenoids have evoked interest in several related fields. Like carotenoids from other groups of organisms, such as plants, algae, and bacteria, carotenoids of fungal origins are known to have characteristic hues of yellow, orange, and red. This is ascribed to the ubiquitous presence, in their molecular structures, of an aliphatic polyene chain comprising eight isoprene units involving light-absorbing conjugated double bonds, the latter of which is responsible for the physicochemical traits of carotenoids [1,20]. 

Some carotenoids act as precursors of vitamin A in mammals including humans, specifically those with β-ring end groups, such as β-carotene, zeaxanthin, and β-cryotoxanthin [21]. Indeed, carotenoids are among the most medically significant bio-pigments. Mounting evidence has revealed that carotenoids can lower the risk of cardiovascular diseases, age-related eye disorders such as macular degeneration and cataracts [22], and cancers [1,21,23].

So far, over 200 fungal species have been documented to be able to produce carotenes [24]. Carotenoid-producing fungi are very diverse, including *Rhodotorula mucilaginosa* [25], *Rhodotorula glutinis* [26], *Blakeslea trispora* [27], *Phycomyces blakesleeanus*, *Mucor circinelloides*, *Fusarium sporotrichioides* [28], *Rhodosporium paludigenum*, *Neurospora crassa*, and *Xanthophyllomyces dendrorhous* [29]. Among them, *Blakeslea trispora* stands out as a major industrial fungus used to produce β-carotene as a colorant in foods, which was authorized by EU Health and Consumer Protection Directorate General in 1995 [24].

*Blakeslea trispora* is a filamentous fungus capable of producing β-carotene through lycopene cyclization [30]. Lycopene is a red-colored intermediate of the β-carotene biosynthesis in *B. trispora* and is converted from isopentenyl pyrophosphate (IPP, C_5_), the first isoprene unit of the terpenoid synthetic pathway [31]. The fungal isopentenyl pyrophosphate (IPP) is produced via the mevalonate (MVA) pathway, with acetyl-CoA as a starting precursor [1,31].

**Figure 1:**
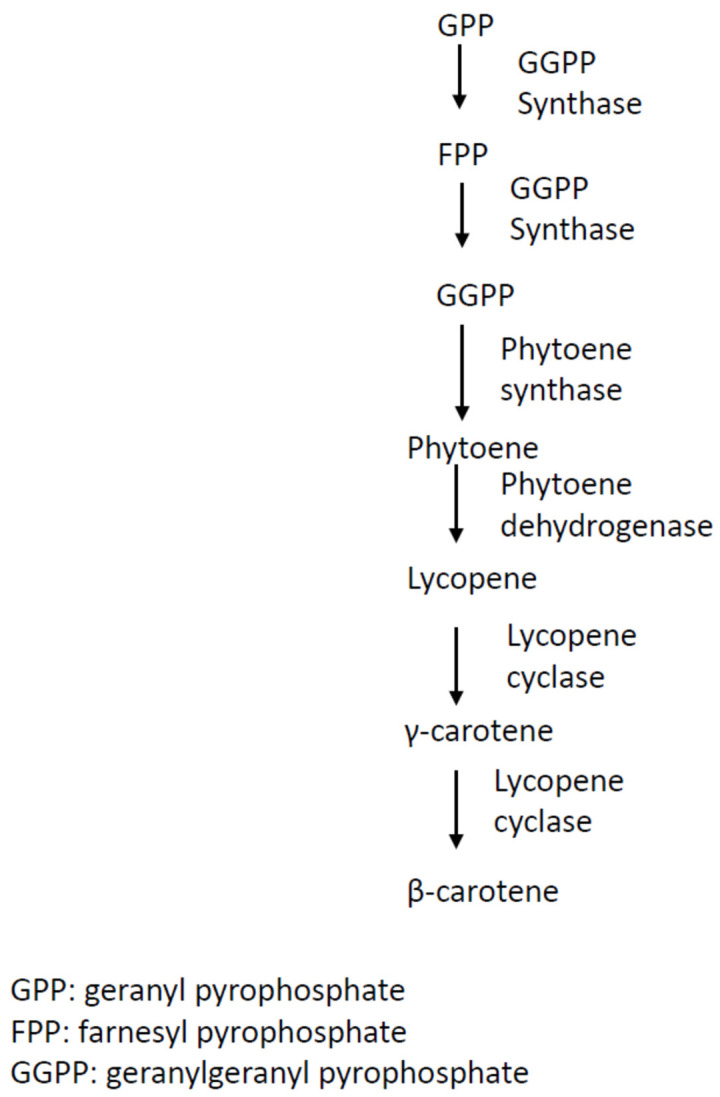
Biosynthesis pathway of β-carotene in *Blakeslea trispora.* This schematic pathway was drawn based on information reviewed in reference [34].

The early biosynthetic steps of β-carotene involve the sequential additions of IPP (C_5_) units to yield geranyl pyrophosphate (GPP, C_10_), farnesyl pyrophosphate (FPP, C_15_), and geranylgeranyl pyrophosphate (GGPP, C_20_) [31]. The initial compound bearing the typical aliphatic carotenoid-like structure is phytoene (a colorless molecule), converted from the condensation reaction of two GGPP units catalyzed by phytoene synthase (Figure 1). The successive step is the dehydrogenase-catalyzed conversion of phytoene to lycopene, followed by the sequential cyclization of lycopene to produce γ-carotene, and then to β-carotene, catalyzed by lycopene cyclase (Figure 1) [32,33]. 

### 2.2. Melanins

Melanins of fungal origin are generated by a complicated polymerization process involving polyketides and free radicals, with the resultant compounds displaying dark green, brown, or black hues. They are resistant to chemical degradation by acids and are insoluble in most solvents; they are only susceptible to degradation by oxidation and are soluble in alkaline solvents [35]. 

Fungal melanins are negatively charged and hydrophobic [36]. They are deposited in fungal cell walls or as extracellular polymers accumulated in the medium around fungal cells. The production and secretion of melanin is associated with fungal resistance against ultra-violet (UV) irradiation, extreme temperature, enzymatic lysis, oxidants, and desiccation; with maintenance of an appropriate balance of metal ions; and with provision of structural rigidity to cell walls [37]. In other words, melanins represent a kind of fungal adaptation strategy for environmental stresses. 

On the other hand, in some mammalian pathogenic fungi, exemplified by *Cryptococcus neoformans*, *Aspergillus fumigatus*, *Candida albicans*, *Candida glabrata*, and *Candida parapsilosis*, melanins might contribute to fungal virulence, reflecting a resistance mechanism of fungal pathogens to the host immune system, such as macrophages, monocytes, and dendritic cells. Recent investigations have revealed that melanin pigments can lead to antigen masking to circumvent the recognition by host immune system, thereby facilitating the survival of pathogenic fungal species against phagocytosis [6,38]. Studies with the dematiaceous (darkly-pigmented) fungus *Wangiella dermatitidis*, which causes a rare but potentially lethal human mycosis, have shown that loss of melanin production might result in the abolishment of invasive hyphal forms, increased vulnerability to neutrophil killing, and attenuated virulence in the murine infection models [39]. 

Apart from their protective role and resistance mechanism towards unfavorable conditions, which renders them promising bio-compounds in medicine and the food industry, fungal melanins are also used in the field of material engineering as a novel biopolymer [40]. Of note, two halophilic fungal species, *Trimmatostroma salinum* and *Phaeotheca triangularis*, isolated from the eastern coast of the Adriatic Sea, have been reported to produce melanin with a supply of saturated sodium chloride [41]. In addition, an Antarctic desert-inhabiting fungus, *Cryomyces antarcticus*, has recently been found to produce melanin pigments possessing high-dose radiation resistance [42]. Collectively, these fungal extremophiles are recognized as prolific sources of melanin with desirable physicochemical properties for industrial and medical applications. 

Given that both pigmented (melanized) and albino strains of the same fungi can survive and grow under laboratory settings, melanins are not essential for fungal growth (primary metabolism). Rather, they are used for fungal defense and resistance (secondary metabolism) against external conditions, including adverse milieu within host, substantial temperature oscillation, extensive radiation exposure, oxidative stress, osmotic pressure (high salinity), desiccation, and poor nutrient supply. 

**Figure 2:**
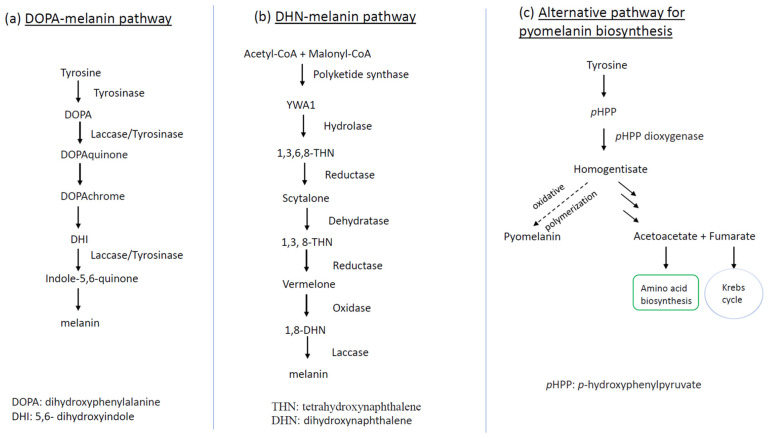
Schematic representation of fungal melanin biosynthetic pathways: (**a**) DOPA-melanin production route as shown in *C. neoformans*; (**b**) biosynthetic pathway of DHN-melanin for deposit in conidial cell wall as manifested by *A. fumigatus*; (**c**) alternative pathway of pyomelanin biosynthesis linked to conidial germination of *A. fumigatus*. The dotted line in (**c**) means that the step is active only during conidial germination. YWA1: Yellow pigment intermediate of WA polyketide synthase.

Recent studies have shown that fungi usually synthesize three different kinds of melanin: cell wall immobilized DOPA (dihydroxyphenylalanine)-melanin, DHN (dihydroxynaphthalene)-melanin, and extracellular water-soluble pyomelanin [Figure 2]. *A. fumigatus* is recognized to be able to produce the latter two, whereas *C. neoformans* can biosynthesize the former one [43]. In *C. neoformans*, DOPA-melanin biosynthesis proceeds through a series of oxidation-reduction reactions, starting from tyrosine or L-3,4 dihydroxyphenylalanine (L-dopa) (see Figure 2a for details). These compounds are subsequently oxidized to DOPA-quinone by a phenol oxidase enzyme such as laccase or tyrosinase, depending on the substrates. The ensuing reactions towards melanin synthesis proceed very quickly, yielding the intermediate dihydroxyindole, which in turn gives rise to melanin via polymerization [44].

Fungi may also synthesize melanin via the DHN pathway, as exemplified by the common mold and opportunistic human pathogen *A. fumigatus*. The DHN-melanin biosynthetic pathway is encoded in a gene cluster consisting of six genes—*abr1*, *abr2*, *ayg1*, *arp1*, *arp2*, and *pksP*/*alb1*―located on its second chromosome [45]. The initial gene in the biosynthetic pathway is *pksP*/*alb1*, which encodes a polyketide synthase (PKS) catalyzing the conversion (β-ketoacyl condensation) from the acetyl-CoA and malonyl-CoA to the heptaketide napthopyrone YWA1. The second step is hydrolysis, catalyzed by the *ayg1*-coding enzyme, which converts YWA1 to 1,3,6,8-tetrahydroxynaphthalene (1,3,6,8-THN). Subsequently, the pathway implements a series of reduction steps followed by aromatization/dehydration, which ends in an oxidative polymerization. Briefly speaking, the resultant intermediate 1,3,6,8-THN is reduced again by Arp2p (Arp2 protein), giving rise to vermelone, the latter of which is oxidized by the copper oxidase Abr1p to form the penultimate product 1,8-DHN. The last reaction generates DHN-melanin from 1,8-DHN, catalyzed by the laccase Abr2p [46].

The other type of melanin generated by *A. fumigatus* is pyomelanin, a pigment with a dark brown hue. In contrast to DHN-melanin, the production of pyomelanin does not have its own biosynthetic pathway but rather proceeds via the polymerization of homogentisate, an intermediate derived from the degradation of L-tyrosine/L-phenylalanine, which is coupled with conidial germination [43,47]. 

Recent studies with the commercial wood-ear mushroom *Auricularia auricula* have shown its melanin in association with the mushroom cell wall, which is analogous to that of the model fungus *C. neoformans*, belonging to DOPA-melanin [48,49]. See Figure 2a for the biosynthesis of melanin in *A. auricula*.

Strikingly, *A. auricula* represents the most feasible commercial melanin producer in terms of scalability and its non-pathogenic feature. It has been proposed that fungal melanins, regardless of their precursors and biosynthetic paths, likely share similar functional groups and analogous physicochemical traits [50]. This black mushroom is found to yield up to 10% melanin in fungal dry mass [48]. *A. auricula* is a popular edible and medicinal mushroom in eastern Asia and is attracting increasing interest from consumers in Europe as well as North America. More recently, *A. auricula* mushroom wastes have been reported as a rich source of melanin for extraction, with an output of up to 11% of fungal dry mass under optimal conditions [51]. Although the industrial process of fungal melanin production is still in its infancy, in-depth explorations with *A. auricula* melanin—biosynthetic steps, fermentation, extraction, and isolation—are promising since this mushroom, in terms of a melanin provider, has several advantages over plant- and animal-derived sources, including independence of seasonal variations, low-cost maintenance, expedient operation, and affordable reaction conditions.

Melanin is widely regarded to possess radioprotective properties due to its ability to scavenge free radicals generated by radiation, and the presence of its numerous aromatic oligomers allowing the dissipation of high-energy recoil electrons [50,52]. It is proposed that melanized fungi isolated from extreme environments, such as the leakage areas around the Chernobyl nuclear power plant and Antarctic highlands, could be used for absorption and decontamination of nuclear waste [53]. 

### 2.3. Polyketides

Polyketide-based pigments of fungal origins are abundantly produced by many fungi, including most filamentous ascomycete genera. Anthraquinones and naphthoquinones are representative classes of polyketide-based pigments in fungi, displaying various colors [1]. 

The pigment class of anthraquinones is characterized by a polycyclic aromatic hydrocarbon structure stemming from the merger of three benzene rings. The diversity of anthraquinones results from the presence of different substituents, such as -OH, -CH_3_, -OCH_3_, and -CH_2_OH, and from the reduction of carbonyl groups as well as double bonds in the benzene ring, allowing the formation of hydroxyanthraquinones. 

Anthraquinones can generate a broad scope of hues, from yellows to reds and even blue shades. Their wide spectrum of colors is attributed to their relatively short conjugated chromophores. They give weak yellows in simple unsubstituted forms, while allowing the attachment of substituents to generate extensive bathochromic shifts of the absorption maximum and, as a result, give reds and even blues.

Anthraquinones including hydroxyanthraquinones (HAQNs) are commonly produced by fungi, such as *Aspergillus* spp., *Eurotium* spp., *Fusarium* spp., *Dreschlera* spp., *Penicillium* spp., *Emericella purpurea*, *Culvularia lunata*, *Mycosphaerella rubella*, and *Microsporum* spp. [1,54]. Of note is Arpink Red™, the polyketide anthraquinone-based natural food colorant initially described in 2004 [55]. This colorant is commercially produced using the fungus *Penicillium oxalicum* var. *Armeniaca*.

Like anthraquinones, naphthoquinone pigments are produced by many fungal species. Most naphthoquinones are colored, usually varying between yellow, orange, and brown [56]. It has been reported that the biosynthesis of naphthoquinone pigments in some *Fusarium* species is triggered by environmental stresses, manifested under the conditions of growth inhibition or arrest [57]. Indeed, many fungal secondary metabolites, such as naphthoquinone pigments, are induced by factors of biotic and abiotic origin, including competition from other organisms, the presence of toxic compounds, etc. [58]. *Penicillium* and the related genus *Talaromyces* can produce naphthoquinone pigments and are widespread, requiring minimal conditions to grow. These fungi are aerobic, require minimal nutrients, and can grow quickly at room temperature. Thus, these genera are promising candidates for the biosynthesis of pigments in laboratory and industrial settings and are particularly relevant to the modulation of biomass or pigment production [9]. 

The synthesis of fungal polyketides requires polyketide synthase (PKS) and uses precursors such as acetyl-CoA and malonyl-CoA [59]. Characteristic polyketide pigments produced by fungi are known to be anthraquinones, hydroxyanthraquinones, and naphthoquinones [59]. 

**Figure 3:**
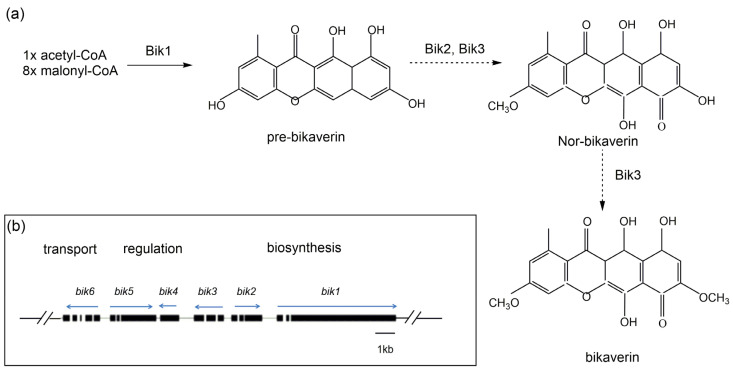
Schematic representation of bikaverin biosynthetic route in *Fusarium fujikuroi*. (**a**) The preferred biosynthetic steps are indicated by bold arrows, and some possible conversions are indicated by dashed arrows. (**b**) Organization of bikaverin (bik) gene cluster in *F. fujikuroi*. Direction of transcription for each predicted gene is indicted by the arrows [62,63].

Bikaverin, belonging to the naphthoquinone class, is a kind of bright red pigment present predominantly in the genus *Fusarium*, among which *Fusarium fujikuroi* is a representative species [60]. In *F. fujikuroi*, bikaverin (viz. 6, 11-dihydroxy-3, 8-dimethoxy-1-methyl-benzo-xanthein-7, 10, 12-trion) is produced via a polyketide biosynthetic pathway (Figure 3a). Genetic analyses with *F. fujikuroi* have demonstrated that a gene cluster composed of six *bik* genes, encoding a multifunctional polyketide synthase, is responsible for bikaverin biosynthesis [61]. Within the *bik* gene cluster situated on chromosome 5 of *F. fujikuroi*, *bik* 1–3 are recognized to be biosynthetic genes, *bik* 4 and *bik* 5 are the regulatory genes, and *bik* 6 encodes a transporter of bikaverin (Figure 3b).

Bikaverin is of great interest due to its pharmaceutical values along with its use as a colorant in the textile industry. Its wide-spectrum bioactivities, including anti-microbial and anti-proliferative properties, make it a promising pigment for further development in pharmaceutical discovery and industrial applications [64]. 

### 2.4. Azaphilones

Azaphilone pigments are a structurally diverse group of fungal pigments. They are polyketide derivates, that is, pigments with pyrone-quinone structures encompassing a highly oxygenated bicyclic core and a chiral quaternary center [65,66,67]. Azaphilones are structurally diverse due to the easy insertion of nitrogen.

The azaphilone-producing fungi are widespread in nature, including microscopic fungi (also called “mold”), such as species in genera *Penicillium*, *Monascus*, *Chaetomium,* and *Talaromyces*, as well as fungi devoid of mold appearance such as those in genera *Hypoxylon*, *Daldinia*, *Creosphaeria* (members of the Xylariaceae family), and *Bulgaria* (Bulgariaceae). Among these genera, *Monascus* is the best known and has provided colorants for human use for over two thousand years. Azaphilone pigments give rise to yellow, red, or green hues of fruiting bodies and/or mycelia.

The genus *Monascus* includes three species: *M. pilosus*, *M. purpureus,* and *M. ruber*. They make up most food colorant strains in Asian food [24]. *Monascus* pigments generally consist of six major azaphilone pigments: ankaflavin and monascin (yellow), monascorubrin and rubropunctatin (orange), as well as monascorubramine and rubropunctamine (violet). However, some strains of *Monascus* spp. can co-produce citrinin, a mycotoxin displaying hepatotoxic and nephrotoxic effects on humans [68]. Hence, traditional food colorants from Asia (the so-called *Monascus* pigments) are banned in the European Union (EU) and the United States, and there have been disputes over their safety when used in food. Strain selection and modification (via genetic manipulation) for citrinin-nonproducing *M. purpureus* is of importance for the commercial production of *Monascus* pigments in the food industry as well as that of monacolin K (viz. lovastatin, an anti-hypercholestrolemia drug) in the pharmaceutical industry. 

This list of azaphilone producers is being extended to include more fungal species. These additional fungi include *Phomopsis* sp. (an endophytic strain producing phomopsones A-C, [69]), *Pseudohalonectria adversaria* (an aquatic fungus producing pseudohalonectrin A-B, [67]), *Aspergillus niger* ATCC 1015 (a type strain of *Aspergillus* producing Azanigerones B-C, [70]), *Aspergillus fumigatus* 14–27 (a gorgonian-derived *Aspergillus* producing pinophilin B, [71]), *Fusarium* sp. (an endophytic fungus producing fusarone, [72]), *Xylariales* sp. PSU-ES163 (seagrass-derived fungus producing xylariphilone [73]), *Coniella fragariae* (goose dung-derived fungus producing coniellins H and I [74]), *Dothideomycete* sp.(an endophytic strain producing austdiol [75]), *Cladosporium perangustm* FS62 (marine sediment-derived fungus producing perangustols A and B, [76]) and *Pleosporales* sp. (a marine-derived fungus producing pleosporalone A [77]).

**Figure 4:**
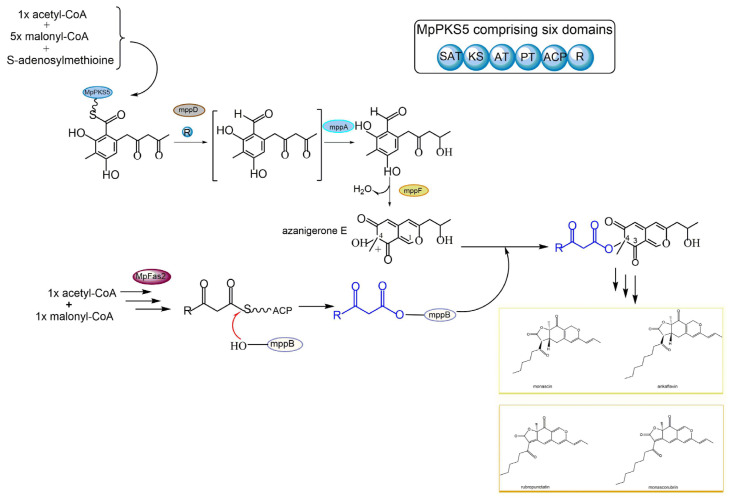
Schematic diagram of various azaphilone pigment biosynthesis via polyketide synthase (PKS) pathway and fatty acid synthase (FAS) pathway in *Monascus purpureus*.

*Monascus* azaphilone pigments (MonAzps) are known to be synthesized via a polyketide pathway, where polyketide synthase (PKS) and fatty acid synthase (FAS) play vital roles [78]. The sophisticated biosynthetic pathway in *Monascus* fungi is considered as a model system to elucidate and dissect the biosynthesis of fungal azaphilone pigments (Figure 4). *M. purpureus* is one of most widely used industrial strains in different areas of China for manufacturing food colorants, while in Japan it is the only authorized species for food use [79]. 

In *Monascus purpureus,* the main pathway of MonAzps biosynthesis starts with a non-ribosome PKS enzyme termed MpPKS5, which consists of six essential domains: acyl carrier protein transacylase (SAT), ketoacyl synthase (KS), acyltransferase (AT), product template (PT), acyl carrier protein (ACP), and reductive release (R) domain (Figure 4). The multifunctional MpPKS5, aided by a serine hydrolase encoded by *mppD*, has been found to produce a highly reactive intermediate, namely hexaketide benzaldehyde. The ensuing step is catalyzed by a ketoreductase (coded by *mppA*), allowing the formation of the corresponding alcohol, namely FK17-P2a, the first stable intermediate in the biosynthetic pathway. FK17-P2a is subsequently oxidized by FAD-dependent monooxygenase (coded by *mppF*) to generate azanigerone E, giving rise to the pyranoquinone skeleton, the so-called polyketide chromophore for azaphilone biosynthesis. In parallel to the above-stated polyketide synthase pathway, MpFas2, the canonical fatty acid synthetase, is also involved in the biosynthesis of MonAzps. MpFas2 has been described to generate short-chain 3-oxo-fatty acyl thioesters, the side chain moiety for *Monascus* azaphilones. The *M. purpureus* biosynthetic gene cluster harbors an O-acyl transferase (coded by *mppB*), which is proposed as the catalyst for the transfer of the MpFas2 products (the side-chain fatty acyl moiety) to the C-4 hydroxyl group. Further steps result in the production of the classical yellow pigments monascin and ankaflavin, as well as orange pigments rubropunctatin and monascorubrin (Figure 4). Together, the main pathway of *Monascus* azaphilone biosynthesis contributes dominantly to the above-described four kinds of classical azaphilone pigments. However, the pyran rings of the classical orange pigments rubropunctatin and monascorubrin are vulnerable to spontaneous O-to-N substitution by readily reacting with endogenous amines to yield the γ-vinylogous pyridines of the red pigments termed rubropunctamine and monascorubramine [80].

Comparative transcriptome studies with *M. purpureus* YY-1 have found that carbon starvation stress, from the use of relatively low-quality carbon sources, might result in high pigment production [78]. The above-mentioned phenomenon was attributed to the suppression of the central carbon metabolism and enhancement of the acetyl-CoA pool, which would provide important substrate to produce azaphilone pigments. Two different carbon sources, soluble starch and glycerol, have been investigated in *M. purpureus* FAFU618 for their effects on production of MonAzPs [81]. The production of intracellular and extracellular pigments apparently differed between the soluble starch group (SSG) and glycerol group (GCG). Moreover, the analyses of intracellular pigments determined by UPLC-QTOF-MS/MS showed significant increases of monascin and ankaflavin in the GCG and the elevation of rubropunctatin and monascorubrin in the SSG. The expression levels of MonAzP biosynthetic genes revealed by RT-qPCR demonstrated that genes *mppA*, *mppC*, *mppR1,* and *mppR2* were down-regulated, while *MpPKS5*, *MpFasA2*, *MpFasB2*, *mppB*, *mppD,* and *mppE* were up-regulated. Meanwhile, the study found 27 differentially expressed proteins of mycelia between SSG and GCG, among which 18 proteins were upregulated, and these proteins were associated with the glycolytic pathway, translation, energy generation, and proteolysis. The remaining nine proteins were downregulated in GCG, and they include ribosomal proteins, heat shock proteins (HSPs), and others. Taken together, their investigation strongly suggests that the control of MonAzPs production is not only closely linked to translational levels of key proteins (enzymes) in the polyketide biosynthetic routes but also tightly associated with the levels of primary metabolism-generated molecules serving as substrates for polyketide synthesis [81].

## 3. Stress Factors and Fungal Pigment Production 

Pigments are often produced to perform ecological roles in response to environmental stresses, such as ultraviolet (UV) light, ionizing radiation, oxidizing agents, nutrient deprivation, hypersaline, and host immunoreactivity. Rather than producing new compounds to build up fungal cells (primary metabolism), these fungi start to biosynthesize secondary metabolites manifested as pigmentation. The yield of the pigments might have originated from the defense demands of the fungi to fulfill the protective role, preventing their mycelia from being hydrolyzed by enzymes produced by other microbes [82]. It could also be a by-product of entering dormancy. In addition, in view of known antioxidant characteristics of these lipophilic pigments, carotenoid production in fungi has been suggested as a natural mechanism to protect against photo-oxidative damage in light-intensive habitats [83,84]. 

In *Fusarium* spp., light has been shown to stimulate the production of carotenoids. In both *Fusarium aquaeductuum* and *F. fujikuroi*, when illumination was supplied in the initially dark-grown culture, carotenoids accumulated for several hours upon light onset, exhibiting a typical orange pigmentation [85,86].

*F. fujikuroi* can produce carotenoids in addition to the phytohormone gibberellin [87] and the polyketide bikaverin [61,88]. Indeed, light induction of carotenoid biosynthesis has been commonly found in the genus *Fusarium*, being first reported in *F. aquaeductuum* in 1969 [89]. Investigation concerning the effects of light on the photoinduction in *F. aquaeductuum* [89] has unveiled that the levels of produced carotenoids depend on the logarithm of the incident light over a 100-fold range, in which the reciprocity law holds true over a broad spectrum of light intensity and time. In the case of *F. aquaeductuum*, the accumulation of carotenoid is induced only by light with a wavelength shorter than 520 nm [89].

Early studies with *F. aquaeductuum* regarding photo-regulated carotenogenesis were subsequently extended to *F. fujikuroi* and *F. oxysporum*. The major carotenoid produced by *Fusarium* spp. is neurosporaxanthin, an acidic apocarotenoid converted from geranylgeranyl pyrophosphate (GGPP) by the sequential activity of four enzymes, encoded by the genes *carRA*, *carB*, *carT,* and *carD*. Upon exposure to light, the mycelia rapidly displayed pigmentation due to the formation of carotenoids, which started to accumulate 1 h after illumination and reached a maximum about 16 h afterwards [86], exhibiting the pattern of photo-induction of carotenoids similar to that of *N. crassa* [90]. It is widely recognized that photoinduction of carotenogenesis in *Fusarium* species is attributed to a transcriptional elevation of most of the structural genes involved in the carotenoid synthesis [91]. 

Fungi often utilize light to perceive high temperature, UV radiation (genotoxic stressor), and the soil/air interface for their survival and proliferation (i.e., spore dispersal). Fungi respond to light by a sophisticated photosensory system consisting mainly of photoreceptors. Exposure to light, especially blue light, is regarded as the major triggering factor for carotenogenesis [92,93]. The blue light receptors in fungi are composed of VIVID (VVD) protein, the white collar complex (WCC), and the cryptochrome (CRY) [94]. The Vvd-null mutant of *N. crassa* was observed to have enhanced carotenoid accumulation as manifested by intensely pigmented mycelia under constant light. In contrast to *N. crassa*, a mutation of VvdA, an orthologue of *N. crassa* Vvd, in *F. fujikuroi*, might result in paler pigmentation. Further studies have unveiled that the VvdA-regulated carotenogenesis in *F. fujikuroi* is a biphasic photo-response, which bursts in the early phase but decays in the subsequent phase [95].

In addition, further investigation in the high-altitude lake ecosystem located in the Nahuel Huapi National Park (Bariloche, Argentina) revealed that living microorganisms have adopted such strategies as the synthesis of antioxidants and UV sunscreen compounds, including carotenoids and mycosporines, to minimize UV-induced damage [96]. Specifically, Libkind et al. reported that: (i) 24 yeast species were recovered, among which at least four represented novel species; and (ii) carotenogenic yeasts dominated in lakes with higher water transparency (more UV exposure). Taken together, the results suggest UV radiation as an important environmental factor eliciting yeast carotenoid pigment production and thereby affecting the microbial community structure in aquatic habitats.

Similarly, oxidative stress has been found to induce increased hypocrellin production in *Shiraia bambusicola*, a well-known pathogenic fungus of bamboo. Hypocrellins, red pigments belonging to the class of perylenequinonoids, are considered as significant photosensitizers, which can serve as potential new-generation photodynamic therapy (PDT) drugs. Hypocrellins, during illumination, display excellent anticancer [97,98,99,100] and antiviral activities [101]. Additionally, hypocrellin has been long used in Chinese folk medicine for the treatment of skin diseases and stomach aches. For example, a recent study by Deng et al. [102] assessed the biomass and hypocrellin biosynthesis of *Shiraia* sp. SUPER-H168 under high-level H_2_O_2_. The results illustrated that hypocrellin yield was improved by approximately 27% and 25% post 72 h incubation with 10 mM and 20 mM H_2_O_2_, respectively. 

Aside from oxidative agents, light can also affect hypocrellin production as well as growth and reproduction of *Shiraia* sp. SUPER-H168. All incubations under different light (white, red, yellow, green, blue, and purple) conditions were beneficial to aerial hyphal growth as compared to darkness. In contrast, all light conditions examined inhibited hypocrellin biosynthesis, with the strongest inhibition displayed by blue light [103]. 

The mycelial culture of *S. bambusicola* without illumination is a biotechnological alternative for hypocrellin production but accompanied by the low yield. Recent investigation using a light/dark shift (24 h/24 h) regime showed that such illumination treatment not only elevated the hypocrellin level in mycelia by 65%, but also promoted its release into the medium, with the highest total hypocrellin production (181.67 mg/L) on day 8, approximately a 73% rise in comparison to the dark controls. Moreover, light/dark shift was observed to elicit the formation of smaller and more compact fungal pellets without any growth retardation of mycelia, pinpointing a novel promising strategy for large-scale production of hypocrellin in mycelium cultures. Furthermore, the light/dark shift regime upregulated the expression of two reactive oxygen species (ROS)-related genes, including NADPH oxidase and cytochrome c peroxidase, and to induce ROS generation. It was also unveiled that ROS might be involved in the upregulation of key genes for hypocrellin biosynthesis and thereby promote hypocrellin production. Collectively, these results provide a molecular basis for deciphering the impacts of light/dark shift on fungal pigment production and for utilizing a tactical approach to increase hypocrellin production in submerged fermentation of *Shiraia* species [104].

More recent studies with filamentous fungi in the genus *Aspergillus* demonstrated that an increase in UV radiation could lead to a reduction in the *Aspergillus* spp. population density in the field (vineyards, nuts, cereal crops). Moreover, it could unbalance the sporulating species present in the field, leading to an elevated prevalence of dark-pigmented conidia [105].

*Monascus* pigments are a representative class of azaphilone pigments, commonly used in Asia since ancient times. Studies with *Monascus pilosus* IFO4520 found that compared to non-illuminated controls, the production of red pigments increased by 1.3-fold after exposure to red light for 7–10 d and displayed a 1.5-fold increase post illumination of 14 d. The above-described findings suggested that red light could stimulate the production of red pigments, especially if applied before stationary growth [106]. Meanwhile, the inhibitory effect of blue light on red pigment production in *M. pilosus* IFO4520 was also observed. Similarly, compared to controls, production of monacolin K (a cholesterol-lowering agent) increased by red light exposure at an early stationary phase. A further investigation with *Monascus* revealed that the reduced pigment accumulation under blue light was due to the increased instability under blue light exposure, even though blue light also stimulated pigment biosynthesis [107]. Interestingly, blue light can exert either inhibitory or stimulatory effects on pigment biosynthesis in Monascus, depending on light intensity and illumination time. Specifically, a study with *Monascus purpureus* strain M9 found that blue light at a low intensity and short exposure time (100 lux, 15 and 30 min/day; 200 lux, 15 min/day) enhanced the production of *Monascus* yellow pigments monascin and ankaflavin, whereas blue light with a high intensity (300 and 450 lux) and long exposure time (45 and 60 min) attenuated pigment production [108].

More recent studies investigated the molecular mechanism underlying blue light-induced pigmentation in *M. purpureus* M9 [109]. During their experimentation, short-term exposure to blue light, namely B15 treatment (blue light exposure for 15 min/d over 8d herein) might lead to oxidative stress in this fungus, manifested by the upregulation in genes encoding cytochrome C peroxidase, peroxidase, etc. In contrast, in response to B15 treatment, there was a reduction in primary metabolic activity, including carbon and nitrogen metabolism. Moreover, metabolic pathways were found to be upregulated involving aromatic amino acids, which yields acetyl-CoA and malonyl-CoA, the precursors for *Monascus* azaphilone pigment biosynthesis. Upon short-term exposure to blue light, *M. purpureus* M9 exhibited a lowered primary metabolism as well as slowed development and reproduction, concomitant with an elevation of secondary metabolism, as evidenced by the enhanced synthesis of photoprotective pigments that absorb blue light. The above-described work represents the initial investigation regarding the genome-wide response of *Monascus* to blue light based on transcriptome sequencing, unveiling that the light induction of *M. purpureus* pigmentogenesis differs remarkably from the well-studied model system observed in *N. crassa* [109].

A previous study with human pathogenic yeast *Malassezia furfur* identified a chemical UV filter called pityriacitrin, a yellow pigment possessing broad absorptive activity in the UV-A, -B and -C regions [110]. *Malassezia*-associated pityriasis versicolor (PV) in humans is known to be characteristic of unexplained color variation and fluorescence of skin lesions. Synthesis of this pigment is related to UV sensitivity in *M. furfur*. The outer part of the human epidermis is the natural habitat of *M. furfur,* and UV exposure is an environmental risk for the fungus (as well as humans; the production of indole alkaloid pigment pityriacitrin in *M. furfur* under the UV radiation may confer this pathogenic yeast certain sun-screening advantages [110]. 

Enhanced growth of melanized fungi has been observed under low dose ionizing radiation, both in the laboratory and in the surroundings of the Chernobyl nuclear plant, suggesting their adaptation to survive or even benefit from ionizing radiation exposure. However, the cellular/molecular mechanism of fungal adaptability in responses to such radiation remains largely unknown. In the model of black yeast *Wangiella dermatitidis*, Robertson et al. demonstrated that ionizing radiation could enhance cell growth by augmenting cell division and cell size [111]. The wild-type (WT) strain and melanin-lacking mutant *wdpks1* of *W. dermatitidis* were investigated under irradiation versus non-irradiation conditions. It was found that long-term exposure to low-dose radiation promoted the survival of both the WT and *wdpks1* mutant. In addition, in association with lowered reactive oxygen species (ROS) levels, there was increased carotenoid production and induction of translesion DNA synthesis. In terms of carotenogenesis, Raman spectral analysis of single cells revealed that ionizing radiation triggered the production of carotenoids in *W. dermatitidis*, with elevated expression of carotenoid biosynthesis genes. This result is reminiscent of the finding by Geis and Szaniszlo [112] that carotenoid syntheses by *W. dermatitidis* in both WT and melanin-deficient (Mel-) strains required photoinduction. The above-described data [111] highlights that participation of an array of cellular components/pathways other than melanin could facilitate *W. dermatitidis* to acclimate to radiation stress for survival and proliferation.

In addition to the light that induces the accumulation of carotenoids in diverse *Fusarium* species, including *F. aquaeductuum* [89], *F. fujikuroi* [86], *F. verticillioides* [95], and *F. oxysporum* [113], another regulatory stressor is nitrogen availability, which can exert a negative effect on carotenogenesis in this fungal genus [60]. Previous work with immobilized mycelia of *F. fujikuroi* incubated under nitrogen limitations revealed an enhanced carotenoid production in comparison to that under an abundant nitrogen supply. Furthermore, the promoting effect of nitrogen deprivation on *F. fujikuroi* carotenogenesis is additive to that induced by light. The photo-induction of carotenoid accumulation is known to act at the transcriptional level, as previously described, whereas the regulation by nitrogen likely involves control of expression at the level of the chromatin structure, as evidenced by the differences in histone methylation found for the genes of the *car* cluster in a *F. graminearum* mutant with impaired methyltransferase KMT6 [114].

Besides light and nitrogen availability, *carS*, encoding a protein of the ring finger family, has been found to be a key regulatory gene affecting *Fusarium* carotenoid biosynthesis. Loss of function of *carS* leads to up-regulation of the structural *car* genes at the transcriptional level [88] and accumulation of high-level carotenoids under different culture conditions [88,115]. 

While the biochemical routes of the carotenoid biosynthesis share many similarities amongst different fungal producers, the regulatory mechanisms often differ, which likely reflects the different demands of different fungal species as finely tuned adaptations to their corresponding habitats in nature [91].

*Monascus* pigments are well-known and described as a mixture of azaphilone compounds, including yellow, orange, and red constituents produced by the Genus *Monascus.* Recent studies with *M. purpureus* YY-1, an extensively used industrial strain in China, have demonstrated that carbon starvation, arising from the supply of relatively low-quality sustained-release carbon sources, may result in the high yield of pigments. The mechanism behind the stimulatory effects of carbon starvation on pigment biosynthesis was proposed to be linked to perturbed central carbon metabolism, thus augmenting the acetyl-CoA pool. Transcriptome studies revealed that carbon starvation stress might cause the downregulation of genes associated with the utilization of glucose from glycolysis to the tricarboxylic acid (TCA) cycle. Altered glycolytic flux might influence the availability of acetyl-CoA, thereby affecting production of secondary metabolites including pigments. The findings offer important insights into the evolution and adaptation of this industrially significant fungus, thereby broadening the avenues for genetic manipulation of this strain for an enhanced pigment production in the future [78].

Previous investigations with *M. purpureus* LPB 97 via solid-state fermentation demonstrated an alteration in pigment production and growth after treatments of high temperature and high-level salt. Specifically, exposure to a temperature over 45 °C was observed to induce the yield of more yellow pigments than untreated controls. A high concentration of NaCl was found to greatly stimulate red pigment production as well as to induce conidiation, but with a significant decrease in biomass [116].

The impacts of temperature and salinity on fungal pigment yield and growth have also been revealed in *Talaromyces albobiverticillius* 30548, a red-pigment-producing isolate derived from the Indian Ocean. The maximal yield of pigment was achieved in a non-salted medium, at 27 °C after 10 d of submerged fermentation in potato dextrose broth. However, maximal biomass production in dry weight was obtained when the medium was supplemented with 9% sea salts at the same temperature. The above results strongly suggest a positive relationship between salinity of the culture and the fungal biomass. Notably, pigment yields decreased with the elevation of salinity over 6%. Furthermore, the color distribution of fungal pigments, illustrated by the CIELAB color system, was found to depend on the salinity in the culture media. This work has unequivocally demonstrated the effects of abiotic (osmotic and thermal) stresses on the pigment production as well as the growth of marine fungi [117].

Microcolonial fungi grown in the rocks under extreme conditions such as at freezing temperatures, drought, strong UV radiation, and poor nutrients are often highly melanized [118,119]. Examples of such fungal extremophiles include black fungi *Cryomyces antarcticus* and *Cryomyces minteri* in Antarctic habitats [120]. Those black fungi can produce thickened melanized cell walls in order to survive the harsh environment, whereby melanin pigments provide protection from the external stressor. 

Apart from cryotolerant species, melanized fungi often possess enhanced resistance to high doses of salts. Fungi such as *Hortaea werneckii, Phaeotheca triangularis, Trimmatostroma salinum, Aureobasidium pullulans*, and *Cladosporium* spp. colonize salterns, and they are capable of withstanding high and even close-to-saturation salt levels. Melanized *H. werneckii* cells could limit the permeability of the cell wall to glycerol, the latter of which functions as a compatible solute under saline stress, thereby reducing the flow of salt into the cell [121].

Intriguingly, sexual hormones like testosterone have effects on the melanin biosynthesis of the human pathogenic fungus *Cryptococcus neoformans*, suggesting the interaction of the host physiochemical milieu and this fungal pathogen. The work also indicated that in vivo melanization of human pathogenic species such as *C. neoformans* is gender-biased [122]. Accordingly, this phenomenon reflects the stress responses of *C. neoformans* towards human physiochemical status while colonizing and surviving within human tissues. 

## 4. Minor Fungal Pigments 

Apart from the major classes of fungal pigments carotenoids, melanin, polyketides, and azaphilones described above, the past decades have characterized an increasing number of minor fungal pigments. These minor pigments have attracted considerable interest from both academia and industry. The notable ones include naphtho-γ-pyrones, sclerotiorin, xanthenes, and xanthones. 

### 4.1. Naphtho-γ-Pyrones

Four novel dimeric naphtho-*γ*-pyrones, namely rubasperone D-F, and an atropisomer rubasperone G, were recently found in marine mangrove fungus *Aspergillus tubingensis* GX1-5E [123]. Pale-red-colored pigment rubasperone D, along with the known naphtho-*γ*-pyrones (monomeric) such as TMC-256A1, rubrofusarin B, and flavasperone, were revealed to possess bioactivity against various cancer cell lines, including breast carcinoma (MCF-7 and MDA-MB-435), hepatoma (Hep3B and Huh7), and glioblastoma (SNB19 and U87 MG). In addition to its antiproliferative activity against human cancers, yellow pigment TMC-256A1 is known as a selective inhibitor of IL-4 signal transduction. IL-4 signaling is essential for mammalian IgE production and development, suggesting its potential as the lead pharmaceutical molecule for the treatment of human allergic diseases [124].

### 4.2. Sclerotiorin 

The orange-colored pigment sclerotiorin has been identified from the culture of *Penicillium sclerotiorum* 2AV2, a fungal strain recovered from Amazonian soil [18]. The authors found an abundance of *Penicillium* sp. comprising the Amazon soil mycobiome where *P. sclerotiorum* 2AV2 was collected, with the remaining fungal species belonging to genera *Aspergillus*, *Trichoderma*, *Fusarium*, and *Paecilomyces*.

### 4.3. Xanthenes

Pigments of the xanthene class, designated ergoflavin and dicerandrol A-C, have been characterized in an endophytic fungus from the leaves of the plant *Mimosops elengi* in India and leaves of an endangered mint (*Dicerandra frutescens*) in USA, respectively [125,126]. Ergoflavin possesses anti-inflammatory activity and anticancer effects towards a number of carcinoma cells, including cells from renal cell carcinoma, pancreatic tumors, non-small-cell lung carcinoma, and colon cancer [125]. Dicerandrols A-C, which are yellow pigments, were demonstrated to have antimicrobial activity against Gram-positive bacteria *Staphylococcus aureus* and *Bacillus subtilis* [126]. In the latter work, dicerandrols A-C, derived from the fungus *Phomopsis longicolla,* endophytic to a different mint plant species grown in central Florida, were found to be structurally related to the ergochromes, which ergoflavin belongs to.

### 4.4. Xanthone

Dihydroxanthene-1,9-dione (i.e., funiculosone) was recently identified from the culture filtrate of *Talaromyces funiculosus* (Trichocomaceae), an endolichenic fungus isolated from lichen thallus of *Diorygma hieroglyphicum* in India [127]. In addition, two analogues designated as mangrovamide J and ravenelin were also characterized. This work represents recently intensifying interests in the field of endolichenic fungi for exploring the novel bioactive metabolites bearing new structures. For example, an endolichenic fungus characterized as *Aspergillus niger* strain Tiegh was isolated from the lichen thallus of *Parmotrema ravum* in India [128]. A novel 6-benzyl-c-pyrone, named aspergyllone, along with some other known compounds such as aurasperones A and D, asperpyrone A, fonsecinone A, etc., were found in this fungal strain. The brown-red pigment aspergyllone showed potent antifungal activity against *Candida parapsilosis,* with an IC_50_ value of 52 μg/mL. This study is reminiscent of an earlier investigation concerning aurasperone F, a yellow pigment of the naphtho-γ-pyrone class, produced by *Aspergillus niger* C-433. Strain C-433 was isolated from grapes collected in the Languedoc-Roussillon region, France [129]. 

## 5. Application of Pigments: Market Analyses and Future Trends

Fungal pigments have been documented for a broad range of potential applications in different industrial sectors. Compared to their initial uses in food, animal feeds, cosmetics, textiles, leather, and pulp and paper industries, there are increasing uses in agrochemical and pharmaceutical industries [130,131]. However, among the diverse fungal pigments and the expanding areas of application, only a few are currently produced at the industrial scale and commercialized for their applications. There is a long journey from the laboratory to the marketplace for most fungal pigments.

Carotenoids have bright orange to reddish colors and have gained popularity in multifaceted fields for decades. Carotenoids of natural origins can be obtained from plants, algae, animals like aphids, and microorganisms, with fungi accounting for a large proportion of microbial sources. Fungal carotenoids are derived from 3-hydroxy-3-methyl-glutaryl-CoA (HMG-CoA) in the mevalonate (MVA) pathway, regulated by sophisticated genetic mechanisms. Previous studies have shown that species of *Mucoromycota* are excellent carotenoid producers. Among *Mucoromycota* species, *Mucor circinelloides* and *Blakeslea trispora* are the two most prominent industrial producers of β-carotene [132,133]. However, other *Mucoromycota* species such as *Mucor hiemalis*, *Mucor rouxii*, *Mucor mucedo*, *Phycomyces blakesleeanus,* and *Umbelopsis isabellina* have also shown great promise [93]. The pioneering biotechnological company that initiated the production of β-carotene at the industrial scale was the Dutch company Gist-brocades (currently DSM) between 1995 and 2001. However, the former Soviet Union launched the production of β-carotene a decade earlier than Gist-brocades [134]. In 2003, the Spanish company Vitatene (now DSM) began the manufacture of lycopene from *Blakeslea trispora* to supply the European market [127]. The global market for carotenoids reached $1.5 billion in 2017 and, at a compound annual growth rate of 5.7%, it is projected to reach up to 2.0 billion by 2026 [135].

Fungal melanin is a major protective constituent against adverse environmental stresses such as UV radiation, oxidation, desiccation, high saline levels, and heavy metals [136]. Melanin has a low solubility in aqueous or organic solvents, which, to some degree, can hamper the extraction procedures for high yield in industrial settings. However, there have been well-established processes for extracting a variety of fungal melanins [14,137]. For example, melanin up to ca. 10% of the dry biomass has been extracted from commercial and waste mushrooms [48,51]. Melanin excels for its biocompatibility and biodegradability traits, together with scavenging properties, metal chelation, and electronic conductance. As a soft biocompatible functional material with antioxidant properties, it can be used for engineering high-performance optoelectronic devices with low impact, such as memory devices, light-emitting diodes, and field-effect transistors [138]. Research in material engineering often involves a multidisciplinary approach, in which the exploitation of natural melanin producers, especially those of melanized fungi, constituting an indispensable part of melanin engineering. 

Anthraquinones are naturally occurring pigments bearing wide-spectrum hues from yellows to reds and even blue shades, mainly due to their relatively short, conjugated chromophores. These pigments have been used as food colorants, laxatives, antimicrobials, anticancer and anti-inflammatory agents, and as textile dyes. Indeed, their outstanding lightfastness, arising from their chemical structure stability, makes them desirable dyes applicable for wool, cotton, and other fibers in the textile industry [139]. For example, fruit bodies of fungi *Cortinarius sanguineus* and *Cortinarius semisanguineus* were used as sources of anthraquinone dyestuffs [139]. Similarly, red anthraquinones produced by *Fusarium oxysporum* and by *Dermocybe sanguinea*, as well as yellow pigments by *Trichoderma virens* and melanins by *Curvularia lunata,* have been reported as promising dyes for fabrics (i.e., wool and silk), with good colorfastness and crocking properties [140,141].

Pigments of other fungal species, such as *M. purpureus*, *Penicillium* spp., *Fusarium* spp., and *Isaria* spp., were demonstrated as excellent biological dyestuffs for tannery [142]. At present, among the diverse anthraquinone-producing fungi, most research has focused on only a small number of species that meet the criteria proposed by Mapari et al. [143]: (1) non-pathogenic to humans; (2) non-toxigenic under a wide variety of manufacturing conditions; and (3) capable of growing and yielding pigments in liquid media. However, the chemical diversity and biological activities of fungal anthraquinones and their derivatives are enormous, especially those derived from untapped habitats like marine [144] and endophytic sources [145].

The utilization history of naphthoquinones has witnessed the extension of their functionality from the original purposes as dyes to medicinal benefits. Over 100 naphthoquinones with a diversity of structures and physicochemical properties have been characterized from more than 60 filamentous fungi [58,146]. These compounds are found to possess fascinating bioactivities, such as antimicrobial, anti-inflammatory, and anticancer activities, due to their propensity of inhibiting respiration and damaging the DNA of pathogens and cancer cells [147]. Anticancer activity has been reported for 1,4-naphthoquinones and their analogues, the former of which are thought to be the most important and widely distributed compounds within the quinone family. The clinical significance of 1,4-naphthoquinones has kindled tremendous research interest in this class of compounds. 

*Monascus* pigments are food colorants that have been widely used in East Asia for centuries [148]. *Monascus spp.* are known to produce three kinds of polyketides: citrinin, red pigments, and monacolin K [149,150,151]. Given that citrinin is nephrotoxic and hepatotoxic in mammals [151], many countries such as the United States, European countries, and Japan have enacted legislation to limit the usage of *Monascus* pigments as food colorants/additives [151,152]. Consequently, for decades, researchers have continued to explore *Monascus* species and strains that do not produce any citrinins or amounts of citrinin below the legislated level [143]. A recent study [153] assessed the effects of different purple rice varieties on the production of citrinin and red pigments by the *M. purpureus* strain CMU002U (UV-mutant). The lowest level of citrinin at 132 ppb was found in the Na variety, which fulfilled the threshold of food regulations in Japan and the European Union. The highest yield of red pigment was achieved using the fermented Doi Muser variety. Taken together, these data suggest that fermented purple rice is a promising candidate to be developed as a safe food supplement. On the other hand, as initially described by Shimizu et al., a polyketide synthase *pksCT* was essential for citrinin biosynthesis in the industrial species *M. purpureus* [68]. Jia et al. [154] removed citrinin in *M. purpureus* by genetic engineering using *Agrobacterium tumefaciens*-mediated transformation to disrupt the polyketide synthase gene *pksCT* in *M. purpureus* SM001. The resultant citrinin-free *pksCT* mutants were also found to yield the same level of *Monascus* pigments as the wild-type strain. The established system was evaluated and showed a high efficiency in *Monascus* strain improvement, thereby paving the road to manipulate the industrial strains for *Monascus* pigment production.

Metabolic regulation may also be used to eliminate the co-production of citrinin during the *Monascus* pigment biosynthesis. The metabolic behaviors of the citrinin and red pigments were studied in *M. ruber* under various amino acid supplementation in liquid media. Citrinin was totally absent in the *M. ruber* culture where histidine was supplied as the sole nitrogen source. The elimination of citrinin was thought to be from its degradation, due to the presence of hydrogen peroxide and the action of peroxidase, the latter two being involved in the assimilation of histidine. Taken together, histidine was suggested to be the most valuable amino acid, since it could lead to the remarkably high production of red pigments, yielding 715 mg/L after 150 h, and almost complete eradication of mycotoxin citrinin [155].

In addition to the above-stated approaches for citrinin-free pigments in *Monascus* species, abundant work has demonstrated that non-*Monascus* species, such as *Talaromyces* species, may be good alternatives for red pigment production at an industrial scale [156]. A novel *Talaromyces* species, namely *T. atroroseus*, was documented to be non-toxicogenic and capable of secreting large amounts of *Monascus* red pigments [156].

Of note, the favorable traits of fungal pigments also make them attractive for cosmetic industries, for instance, by serving as active ingredients/additives in sunscreen/sunblock facial creams and body lotions, as well as in anti-aging facials/masks [157]. Carotenoid pigments, including astaxanthins, canthaxanthins, carotenes, and lycopene, produced by *B. trispora* and *Phaffia rhodozyma*, have anti-oxidative and photoprotective properties. Melanins from *Aspergillus nidulans* and *Alternaria alternata* possess antimicrobial, antioxidative, and UV-light protective activities. Cosmetic companies such as NINGXIA R.D. and L’OREAL S.A. have licenses for using *Monascus* spp. pigments and *Monascus*-like pigments for manufacturing skin conditioning and skincare products targeting the Asian market [158]. Red (rubropunctamine) and yellow (monascin, ankaflavin) pigments produced by *M. purpureus* NMCCPF01 are naturally occurring sun protection factor (SPF) enhancers when supplementing commercial sunscreen lotions and creams as well as aloe vera extracts [159]. These above-described pigments were found to have antioxidant activity based on DPPH (1,1-Diphenyl-2-picrylhydrazyl) radical scavenging assay and ferric reduction potentials, and to be safe based on human keratinocyte and erythrocyte cytotoxicity assays. The SPF of commercial sunscreens exhibited an increase of 36.5% with red pigment supplementation in contrast to the 13% increase by yellow pigment addition. Thus, pigments from *M. purpureus* may serve as promising candidates to improve the quality of commercial sunscreen products.

Natural colorants, especially hydroxyanthraquinones such as emodin, dermocybin, and dermorubin, including those produced by the ectomycorrhizal fungus *Dermocybe sanguinea,* have been reported as potential hair dyes for coloring human hair [160]. In addition, there are increasing strains and species of marine fungi being exploited for anti-ageing, anti-acne, and skin-tone-adjusting agents, which represent an important future trend of fungal pigments as cosmeceuticals [157,161]. 

While there has been a long history of use of fungal-pigmented wood in intarsia and marquetry craftworks, the use of fungal-pigmented wood for personal aesthetics is a relatively recent phenomenon. However, the application of wood decay fungi as coloring agents to wood, so-called spalting, represents a novel means to increase forestry revenue in a sustainable and green way, particularly prevalent in the Pacific Northwest of North America, which includes parts of the United States and Canada [162,163,164]. Indeed, this is an exciting area of development for decorative wood products and the wood coating industry.

Spalting fungi are a selection of soft-rotting ascomycetes that have been found to stain a variety of materials, including wood [162,163,165], bamboo [166], and textiles [167,168]. The pigments derived from spalting fungi possess such traits as resistance to UV exposure, color fastness, and light fastness under unmordanted conditions, which render them potential novel applications in the wood, painting and textile industry [164,169]. At present, the following fungi and their pigments have attracted significant academic and applied interests: *Scytalidium cuboideum*, which produces a red pigment draconin red [167]; *Scytalidium ganodermophthorum,* producing an undefined yellow pigment [170]; and *Chlorociboria aeruginosa* (Oeder), producing xylindein, a blue-green pigment. 

Xylindein, a naphthoquinone fungal pigment with enhanced stability, was recently reported as a promising material for the organic semiconductor industry. This pigment was demonstrated to possess high photostability, electron mobility up to 0.4 cm^2^/(V s) in amorphous films, and thermally activated charge transport and photo-response with activation energies of ∼0.3 and 0.2 eV, respectively [171,172]. The excellent optical and electronic properties of xylindein solutions and films was found to be attributed to the presence of xylindein tautomers and aggregates [172]. This finding helps shed light on the exploitation of biocompatible and sustainable organic (opto) electronics derived from fungal pigments in the field of material engineering.

Nature offers the fascinating source of safe colors and lead biomolecules for drug discovery. However, limitations such as season-dependent raw material availability and variation in pigment profile pertaining to the colorants of plant origin cannot be ignored in comparison to those of fungal origin. As shown above, pigmented fungi are an enormous compound reservoir for a variety of pigments, including those that are absent in plants such as azaphilones [134]. 

The main challenge of large-scale production of fungal pigments lies in the difficulty in increasing the yield of desirable pigment(s) while minimizing the production of unwanted substances. However, with the rapid advancement in genetic and metabolic engineering, such major biological traits would be addressed with respect to maintaining the robustness of fungal strains and to eliciting higher pigment yields. Selection of prolific fungal strains without mycotoxin co-production and a lowered energy input are the key factors determining market significance. *Monascus* pigments produced by *Monascus* species without citrinin represent a promising area, awaiting legislative approval in the US and the European Union. To ensure their safe supplementation in foods, such pigments must undergo lengthy toxicology testing in line with legislation and regulations in each country or area. 

Furthermore, the functionalities of fungal pigments other than dyeing power, for instance, antioxidant, antimicrobial, anticancer, anti-inflammatory, and immunomodulatory properties, have stimulated dramatic research interests in such compounds, as they have the potential to be lead biomolecules in the discovery and development of pharmaceuticals to treat human diseases, including cancer, infection, and metabolic disorders. 

Fungal pigments are commonly bioactive molecules produced mainly in response to stress conditions, and a large proportion of pigment production is found in isolates recovered from stressful environments such as ice samples, aqueous environments with high salinity, and remote glacial areas in Antarctica, pinpointing fungal pigmentation as an adaptative strategy to unfavorable conditions, i.e., cold and high-UV environments [173]. In this context, untapped habitats in Antarctica and marine environments are potentially rich niches for bioprospecting pigment-producing fungi in the future. The next decades will witness more and more pigment production using fungal cell factories. Such developments will be driven by discoveries of novel strains and new pigments, as well as new cultivation and downstream processing techniques to produce high-quantity and high-quality pigments, as well as new areas of applications of these fungal pigments.

## Data Availability

This is a review article. No new original data was presented in this manuscript.
